# A Case of Guillain-Barré Syndrome Possibly Associated With COVID-19 Infection

**DOI:** 10.7759/cureus.82954

**Published:** 2025-04-24

**Authors:** Rafail Giannas, Louiza Klironomou, Evgenia Skafida

**Affiliations:** 1 Internal Medicine, Syros General Hospital, Ermoupolis, GRC; 2 Nursing, National and Kapodistrian University of Athens, Syros General Hospital, Ermoupolis, GRC; 3 Rheumatology, Asklepieion Hospital, Athens, GRC

**Keywords:** acute inflammatory demyelinating polyneuropathy (aidp), covid-19, electrodiagnostic study, guillain-barre syndrome (gbs), intravenous immunoglobulin (ivig)

## Abstract

Guillain-Barré syndrome (GBS) is a neurological disorder characterized by progressive and symmetric muscle weakness accompanied by the absence or depression of deep tendon reflexes. It is typically associated with a preceding viral infection, such as the Epstein-Barr virus, cytomegalovirus, hepatitis E virus, and Zika virus, or bacterial pathogens like *Campylobacter jejuni* and *Mycoplasma pneumoniae.* Recently, rare cases of GBS have been reported following infection with COVID-19, suggesting a potential association between SARS-CoV-2 and GBS. In this report, we present a clinically and diagnostically confirmed case of GBS following COVID-19 reinfection, which supports emerging data on the neurological complications associated with subsequent episodes of infection by the novel coronavirus.

## Introduction

Guillain-Barré syndrome (GBS) is an acute, immune-mediated inflammatory disorder that affects the peripheral nervous system. It manifests as progressive, symmetrical ascending muscle weakness, reduced or absent deep tendon reflexes, paresthesia, facial muscle involvement, autonomic dysfunction with fluctuations in blood pressure and heart rate, and ophthalmoplegia. In the most severe cases, respiratory muscle involvement may occur, necessitating mechanical ventilation. The syndrome is classified into variants, the most common of which are acute inflammatory demyelinating polyneuropathy (AIDP), acute motor axonal neuropathy, acute motor and sensory axonal neuropathy, Miller Fisher syndrome presenting with ophthalmoplegia, ataxia and areflexia, and Bickerstaff brainstem encephalitis, characterized by altered consciousness, hyperreflexia, and cranial nerve involvement [1]. Typically, GBS is characterized by ascending paralysis, reduced conduction velocities electrophysiologically as well as albuminocytological dissociation based on cerebrospinal fluid (CSF) analysis [2].

GBS is often triggered by specific viral or bacterial infections. It commonly follows a C*ampylobacter jejuni*, cytomegalovirus, Epstein-Barr, or *Mycoplasma pneumoniae* infection, as these pathogens are believed to trigger an aberrant immune response through molecular mimicry, leading to demyelination or axonal damage of peripheral nerves [3]. Patients with COVID‐19 can present neurological symptoms due to either viral neurological damage or indirect neuroinflammatory and autoimmune processes [4]. Although the currently available evidence remains sparse, with some analyses stating that the incidence of GBS has even decreased during the pandemic period [5], emerging case reports and observational studies highlight a possible immune-mediated response in post-COVID-19 cases [6]. This case report aims to contribute to the existing literature on the association of these two entities, especially given the patient's recent first Sars-CoV-2 infection, three months prior, without any sequellae.

It is also important that clinicians maintain a high level of suspicion when evaluating patients with recent COVID-19 who present with neuromuscular symptoms. Early recognition and prompt initiation of treatment, typically with intravenous immunoglobulin (IVIG) or plasma exchange, are crucial in reducing morbidity and improving outcomes.

## Case presentation

A 70-year-old male patient presented to our hospital's emergency department with a progressively worsening history of myalgias, impaired temperature sensation, numbness in both upper and lower limbs, and a headache over the past three days. He had tested positive for COVID-19 via a self-test 24 hours prior and reported a previous COVID-19 infection three months earlier, which was mildly symptomatic and uncomplicated and did not require hospitalization or an antiviral regimen. His medical history included hypertension, for which he was on valsartan/hydrochlorothiazide therapy. The patient reported no history of preexisting neurologic conditions and had concluded his vaccinations a year prior.

Upon examination, his vital signs were as follows: blood pressure of 160/100 mmHg, heart rate of 77 BPM, and oxygen saturation of 99% on room air, and he was afebrile. A neurological evaluation revealed negative Romberg, Unterberger, and Barré tests, as well as a normal mental status. The patient reported generalized dysesthesia and a sensation of heat in his limbs and torso. Upper limb postural tremor and lower limb paresis, with muscle strength graded as 3/5 according to the Medical Research Council (MRC) scale, were also observed, and the patient was initially able to walk less than 10 meters with the use of help [7].

**Figure 1:**
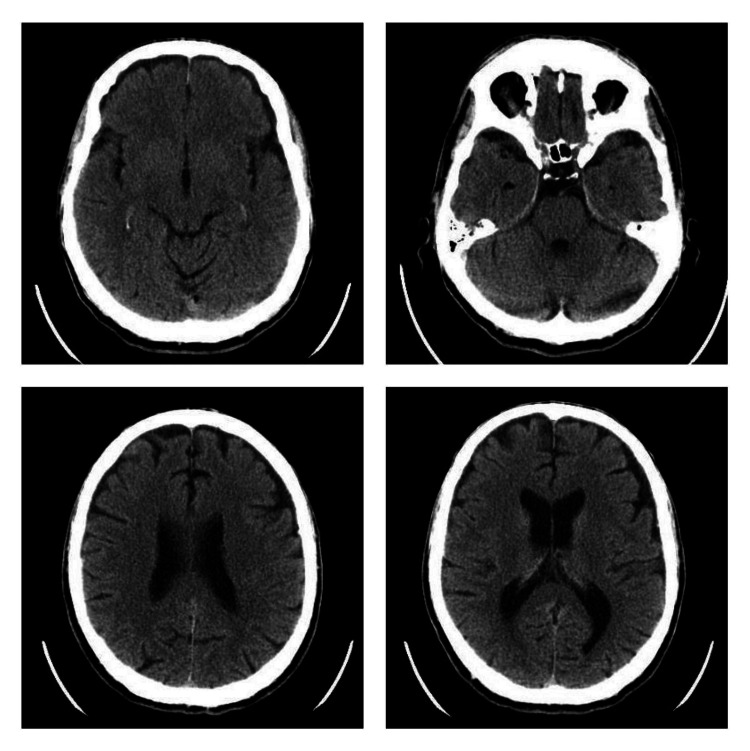
Images from the brain CT scan of the patient demonstrating no abnormalities CT: computed tomography

Routine blood tests showed no significant abnormalities, and stool, urine, and blood cultures were negative. Arterial blood gas analysis revealed mild alkalosis (pH, 7.59; pCO₂, 24 mmHg; HCO₃⁻, 27 mmol/L). A brain computed tomography (CT) scan showed no pathological findings (Figure 1). A key clinical finding was the absence of tendon reflexes in both upper and lower limbs, accompanied by progressive lower limb paresis that rendered him unable to walk by the second day of hospitalization. A lumbar puncture was performed, leading to a probable diagnosis of GBS. The patient was referred to a higher-level neurological center for further evaluation and management. 

At the referral hospital, an electrodiagnostic study performed on the third day of the patient's hospitalization revealed reduced motor and sensory nerve conduction velocities, consistent with demyelinating polyneuropathy primarily affecting motor nerves. The electrodiagnostic study results are summarized in Table 1.

**Table 1:** Electrodiagnostic study performed at the hospital of referral showing reduced motor and sensory nerve conduction velocities, consistent with demyelinating polyneuropathy primarily affecting motor nerves Data for normal range values are from the American Association of Neuromuscular and Electrodiagnostic Medicine (AANEM), reference values, 2020

Motor nerve conduction		Latency (ms)	Normal range (ms)	Amplitude (mV)	Normal range (mV)	Nerve conduction velocity (m/s)	Normal range (m/s)
Upper limbs							
Median nerve	Right	6.17	<4.5	8.3/7.3	>4.1	32.9	>52
	Left	6.46		7.4/5.5		32.1	
Ulnar nerve	Right	5.1	<3.7	5.7/3.1	>7.9	35.3	>52
	Left	5.29		5.8/3.0		32.1	
Lower limbs							
Peroneal nerve	Right	6.29	<6.5	5.7/3.1	>1.3	35.3	>46.5
	Left	7.19		5.8/3.0		32.1	
Tibial nerve	Right	10.4	<6.1	4.7/2.5	>4.4	34.4	>46.6
	Left	8.02		4.5/1.9		32.6	
Sensory nerve conduction		Latency (ms)	Normal range (ms)	Amplitude (μV)	Normal range (μV)	Nerve conduction velocity (m/s)	Normal range (m/s)
Upper limbs							
Median nerve	Right	4.18	<3.3	3.8	>11	35.9	>50
	Left	4.26		11.9		30.5	
Ulnar nerve	Right	4.63	<3.1	15	>10	28.1	>50
	Left	6.08		13		21.4	
Lower limbs							
Sural nerve	Right	2.91	<3.6	4.8	>4	37.8	>48
	Left	3.63		10.7		30.3	

A second lumbar puncture, which was performed 10 days after his initial admission, showed findings suggestive of albuminocytologic dissociation. The results of both lumbar punctures are presented in Table 2.

**Table 2:** Analyses of the patient's lumbar punctures on the day of admission and 10 days later

Cerebrospinal fluid	White blood cell number/mm3	Red blood cell number	Glucose mg/dl (normal <80)	Protein mg/dl (normal <60)	Gram staining
Day 1	1	Rare	71	45	Negative
Day 10	3	Rare	62	88.8	Negative

Further investigations, including tests for HBsAg, anti-HCV, HIV I+II, influenza A1/A2, influenza B, respiratory syncytial virus (RSV), antiganglioside antibodies, carcinoembryonic antigen (CEA), α-FP, Ca 19-9, and prostate-specific antigen (PSA), were all negative. Polymerase chain reaction (PCR) for SARS-CoV-2 was performed 10 days after admission and returned negative. The patient received a five-day intravenous IVIG treatment with a standard single dose (0.4 g/kg bodyweight for five days), with significant improvement in tetraparesis [8]. He was subsequently transferred back to our hospital for physiotherapy and rehabilitation, where further mobility improvement was observed. 

## Discussion

GBS is a rare, debilitating, and potentially life-threatening neurological disorder characterized by inflammation affecting the peripheral nervous system. As previously described, GBS presents in several clinical variants, each with differing neurological manifestations and severity [9]. In this case, the patient’s presentation and electrodiagnostic findings were consistent with the AIDP subtype. Early diagnosis allows for prompt immunotherapy, potentially reducing complications and long-term disability.

The Brighton Collaboration has established diagnostic criteria for GBS [10], including bilateral and flaccid limb weakness, decreased or absent deep tendon reflexes in affected limbs, monophasic disease course with symptom progression reaching a nadir within 12 hours to 28 days, CSF cell count of <50/μl, elevated CSF protein concentration, nerve conduction study findings consistent with GBS subtypes as well as the absence of an alternative diagnosis for weakness [11]. According to these criteria, our patient was diagnosed with GBS, with findings indicative of AIDP predominantly affecting motor nerves.

The differential diagnosis of acute flaccid paralysis includes paralytic poliomyelitis, GBS, and transverse myelitis. Less common etiologies are traumatic neuritis, encephalitis, meningitis, and tumors [12]. The diagnostic process is refined through negative imaging studies, comprehensive clinical evaluation, and close observation of symptom evolution. In this case, a recent history of antecedent infection, absence of trauma, lack of cervical spine rigidity or photophobia, and the patient’s afebrile status throughout hospitalization support the exclusion of other causes and increase the clinical suspicion for GBS.

GBS is recognized as a post-infectious syndrome, with rare reports following COVID-19. It has been linked to several viral and bacterial pathogens [3]. Additionally, some vaccinations have been associated with GBS [3]. Since the emergence of the COVID-19 pandemic, sporadic cases of post-COVID-19 GBS have been reported [13]. Notably, a 2021 study identified 11 cases of GBS among 71,904 COVID-19 patients, yielding an incidence rate of 0.15 per 1,000 COVID-19 cases, a rate significantly higher than the baseline incidence of 0.02 per 1,000 in the general population [13-15].

The mechanisms leading up to COVID-19-driven GBS are speculated to be multifactorial; the most recent molecular studies suggest direct neurotropism and neurovirulence, microvascular dysfunction and oxidative stress, immune system disruption, molecular mimicry, and autoantibody production as the main drivers behind the clinical phenotype [4]. On this ground, it remains a subject of ongoing molecular studies. It is noteworthy that in our case, GBS did not occur as a complication of the first COVID-19 infection.

Public health-wise, recognizing GBS in post-COVID cases helps monitor rare but serious post-infectious outcomes of SARS-CoV-2, supporting vaccine safety surveillance, healthcare preparedness, and guiding rehabilitation services. Awareness can also prevent misdiagnosis, particularly in resource-limited settings where neurological symptoms may be attributed to other causes.

One of the strengths of this report is the timely and comprehensive diagnostic workup, which included neurophysiological studies and CSF analysis, enabling a diagnosis. Furthermore, the clear temporal relationship between SARS-CoV-2 infection and the onset of neurological symptoms supports a plausible post-infectious autoimmune mechanism. However, as a single case report, this study inherently carries limitations; a single case cannot establish causality, nor can it determine incidence or prevalence. Additionally, other contributing factors, such as subclinical co-infections or undetected autoimmune predispositions, cannot be entirely excluded. Nonetheless, given the increasing reports of post-COVID-19 GBS, case reports like this remain valuable for hypothesis generation and for raising clinical awareness of potentially serious post-infectious complications in COVID-19 patients.

## Conclusions

This case report aims to help first aid clinicians to timely recognize a potentially life-threatening disease by describing the hallmark symptoms and typical findings of GBS, since early intervention is crucial in some cases. Secondly, it emphasizes the need for heightened clinical awareness of GBS as a potential complication following COVID-19. As evidence is sparse, there is room for further clarification of the dominant pathophysiological mechanisms leading to GBS, especially in patients with a recent history of mild first episode of COVID-19 infection, which may support the need for a personalized therapeutic approach in such cases, as opposed to a generalized treatment strategy.
